# Carfilzomib, lenalidomide and dexamethasone followed by a second ASCT is an effective strategy in first relapse multiple myeloma: a study on behalf of the Chronic malignancies working party of the EBMT

**DOI:** 10.1038/s41409-023-02048-7

**Published:** 2023-08-05

**Authors:** Rémi Tilmont, Ibrahim Yakoub-Agha, Diderik-Jan Eikema, Nienke Zinger, Mathias Haenel, Nicolaas Schaap, Concepcion Herrera Arroyo, Christine Schuermans, Britta Besemer, Monika Engelhardt, Jürgen Kuball, Mariagrazia Michieli, Natalie Schub, Keith M. O. Wilson, Jean Henri Bourhis, Maria Victoria Mateos, Neil Rabin, Edgar Jost, Nicolaus Kröger, José M Moraleda, Tommaso Za, Patrick J. Hayden, Meral Beksac, Donal Mclornan, Stefan Schönland, Salomon Manier

**Affiliations:** 1 Hematologie Clinique, CHU de Lille, Lille, France; 2 CHU de Lille, Univ Lille, INSERM U1286, Infinite, Lille, France; 3 EBMT Statistical Unit, Leiden, Netherlands; 4 EBMT Leiden Study Unit, Leiden, Netherlands; 5 Klinikum Chemnitz gGmbH, Chemnitz, Germany; 6 Radboud University, Medical Centre Nijmegen, Nijmegen, Netherlands; 7 Hosp. Reina Sofia, Cordoba, Spain; 8 GZA Hospitals, Antwerp, Belgium; 9 Universitaet Tuebingen, Tuebingen, Germany; 10 University of Freiburg, Faculty of Freiburg, Freiburg, Germany; 11 University Medical Centre, Utrecht, Netherlands; 12 Centro di Riferimento Oncologico, Aviano, Italy; 13 University Medical Center Schleswig-Holstein, Campus Kiel, Kiel, Germany; 14 Department of Haematology, Cardiff, UK; 15 Gustave Roussy Cancer Campus, Villejuif, France; 16 Hospital Clínico, Salamanca, Spain; 17 University College London Hospital, London, UK; 18 University Hospital Aachen, Aachen, Germany; 19 University Hospital Eppendorf, Hamburg, Germany; 20 Hospital Universitario Virgen de la Arrixaca, Murcia, Spain; 21 Section of Hematology, Catholic University, Fondazione Policlinico Universitario A. Gemelli IRCCS, Rome, Italy; 22 Department of Haematology, School of Medicine, Trinity College Dublin, Dublin, Ireland; 23 Ankara University Faculty of Medicine, Ankara, Turkey; 24 University College London Hospitals NHS Trust, Heidelberg, Germany; 25 Medizinische Klinik u. Poliklinik V, University of Heidelberg, Heidelberg, Germany; 26 Univ Lille, Canther, INSERM UMR-S1277 CNRS UMR9020, Lille, France

**Keywords:** Myeloma, Stem-cell therapies, Immunotherapy

## Abstract

In the setting of a first relapse of multiple myeloma (MM), a second autologous stem cell transplant (ASCT) following carfilzomib-lenalidomide-dexamethasone (KRd) is an option, although there is scarce data concerning this approach. We performed a retrospective study involving 22 EBMT-affiliated centers. Eligible MM patients had received a second-line treatment with KRd induction followed by a second ASCT between 2016 and 2018. Primary objective was to estimate progression-free survival (PFS) and overall survival (OS). Secondary objectives were to assess the response rate and identify significant variables affecting PFS and OS. Fifty-one patients were identified, with a median age of 62 years. Median PFS after ASCT was 29.5 months while 24- and 36-months OS rates were 92.1% and 84.5%, respectively. Variables affecting PFS were an interval over four years between transplants and the achievement of a very good partial response (VGPR) or better before the relapse ASCT. Our study suggests that a relapse treatment with ASCT after KRd induction is an effective strategy for patients with a lenalidomide-sensitive first relapse. Patients with at least four years of remission after a frontline ASCT and who achieved at least a VGPR after KRd induction appear to benefit the most from this approach.

## Introduction

Multiple myeloma (MM) remains an incurable hematological malignancy despite many recent therapeutic advances [1]. A number of treatment options are now available for refractory/relapsed multiple myeloma (RRMM) and the wide range of possibilities renders decision-making increasingly complex [2]. There remains no standard approach as patients with RRMM display different disease trajectories, with some being primary refractory, others having early relapse, and others undergoing late relapse after autologous stem cell transplantation (ASCT). In addition, MM is a heterogeneous disease with varying biological and molecular characteristics [3]. Based on the International Myeloma Working Group (IMWG) consensus, three cytogenetic abnormalities are associated with an unfavorable prognosis: t(4;14)(p16;q32), t(14;16)(q32;q23) and del17p [4, 5].

In the context of a lenalidomide-sensitive relapse, one of the preferred therapeutic options recommended in the European Hematology Association/European Society of Medical Oncology (EHA/ESMO) and IMWG guidelines is the combination of carfilzomib, lenalidomide and dexamethasone (KRd) [6]. Carfilzomib is a second-generation proteasome inhibitor that has been approved by the European Medicines Agency (EMA) for use in adults who have received at least one previous treatment in combination with lenalidomide and dexamethasone based on data from the ASPIRE trial [7]. This study showed a progression-free survival (PFS) of 26.3 months for KRd *vs*. 17.6 months for Rd (Hazard Ratio (HR) = 0.69, *p* = 0.0001) [8]. In younger, fitter patients, a second salvage ASCT remains an option in the setting of a durable remission following upfront ASCT. The American Society for Transplantation and Cellular Therapy (ASTCT), the European Society for Blood and Marrow Transplantation (EBMT), and IMWG recommend consideration of a second salvage ASCT in patients with a treatment interval of more than 18 months, while the EHA/ESMO recommend an interval of 36 months if patients have received maintenance therapy [6, 9]. Patients with high-risk cytogenetics have been reported to benefit from this procedure as well [10].

There is no consensus on the best first relapse treatment prior to a second ASCT [11]. Based on retrospective data, the most common therapeutic combinations used over the last 15 years contain a proteasome inhibitor [12]. It is generally recommended to combine a PI with an immunomodulatory drug in cytogenetically high-risk MM [13]. Since 2015, KRd followed by an ASCT has been used in MM patients following a first relapse in many European centers, but there remains limited data on patient outcomes. Therefore, we report here characteristics and outcomes from a retrospective multicenter, EBMT registry-based study of 51 patients with MM who received KRd induction followed by a second ASCT following first relapse.

## Methods

### Study design and data collection

This was a retrospective, multicenter, registry-based analysis of transplants performed in centers affiliated to the EBMT. Eligible MM patients had undergone a second line of treatment with KRd induction followed by a second ASCT between January 2016 and December 2018. Patients who received fewer than two cycles of KRd were excluded. Clinical data were collected using ProMISe (Project Manager Internet Server), the international database coordinated by EBMT. Patient outcome data were collected on-site using individual patient records. A total of 51 patients from 22 EBMT registered transplant units were included in the overall analysis. This study was conducted in accordance with the Declaration of Helsinki. All patients gave informed consent for the retrospective use of their clinical data.

### Objectives

The primary objective was to estimate the PFS and overall survival (OS) in patients who received a second line of treatment with KRd followed by a second ASCT. Secondary objectives were to assess the overall response rates (ORR), defined by achievement of partial response (PR) or better and identify variables significantly affecting PFS and OS in this population.

### Treatments

Carfilzomib, lenalidomide, and dexamethasone (KRd) were administered in 28-day cycles. The total number of cycles and the details of treatment administration varied between centers. Carfilzomib was administered intravenously, either twice weekly (D1, D2, D8, D9, D15 and D16) at a dose of 20 mg on D1 and D2 of cycle 1 and 27 mg/m² for each infusion thereafter, or weekly (D1, D8, D15) at a dose of 20 mg on D1 of cycle 1 and 56 mg/m² for each infusion thereafter. Lenalidomide was given orally from D1 to D21 at a dose of 25 mg once daily. Dexamethasone was administered orally at 20 mg twice weekly (D1, D2, D8, D9, D15, D16, D21, D22) or 40 mg weekly (D1, D8, D15, D22). The follow-up cut-off date was April 5th 2021. Hematopoietic stem cells were thawed and re-infused based on local standard operating procedures and according to Joint accreditation committee for ISCT and EBMT (JACIE) recommendations in operation at the time of transplantation.

### Evaluation of endpoints

Diagnostic criteria, the treatment response, and relapse definitions were based on the 2014 IMWG guidelines [5]. OS was measured from Day 0 of the second ASCT until death, regardless of cause. PFS was measured from Day 0 of the second ASCT until relapse or death, regardless of cause. OS and PFS were censored at most recent follow-up. ISS stage and the definition of cytogenetically high-risk disease (presence of del(17p), t(4;14), or t(14;16)) were based on the 2014 IMWG criteria.

### Statistics

Categorical variables were reported as numbers (percentage) and quantitative variables as medians [interquartile range]. Overall survival (OS) and progression-free survival (PFS) functions, censored on the study end date, were estimated by the Kaplan-Meier method. The median follow-up was estimated by means of the reverse Kaplan-Meier method. To compare survival distributions in univariate analysis, the log-rank test and the Cox model were used. When hazards were not proportional between groups (i.e. crossing survival curves), a multiple-direction log-rank test (mdir) with 10,000 iterations was used instead [14, 15]. Two-tailed statistical tests were used. The p values were considered as significant at the 5% level. Data were analyzed with R software, version 4.1.3 [16].

## Results

### Description of the population

A total of 51 patients who received a first relapse treatment with KRd followed by a second ASCT were included in the analysis. Patient characteristics at relapse are shown in Table 1. There were 35 (68.6%) men and the median age was 62 years (IQR 58–66). ISS stage at diagnosis was stage I in 18 patients (41.9%), stage II in 11 (25.6%), stage III in 14 (32.6%) and missing in 8 patients. The monoclonal component was IgG in 29 patients (56.9%), IgA in 12 patients (23.5%) and light chain alone in ten patients (19.6%). Twenty-seven patients (52.9%) had standard-risk cytogenetic profiles and 11 patients (21.6%) had high-risk cytogenetic profiles according to IMWG criteria because they had either a del(17p): 6/11 showed a del(17p) and 5/11 a t(4;14). No t(14;16) was detected in the population and data was missing for 13 patients. Frontline induction was bortezomib, cyclophosphamide, dexamethasone (VCd) in 16 patients (31.4%), bortezomib, adriamycin, dexamethasone (VAD) in 13 patients (25.5%), bortezomib, dexamethasone (Vd) in 7 patients (13.6%), bortezomib, thalidomide, dexamethasone (VTd) in 7 patients (13.6%), bortezomib, lenalidomide, dexamethasone (VRd) in 2 patients (3.9%) and 4 patients received others regimens (7.8%). All patients (51/51) received a frontline ASCT, conditioned with melphalan alone. Five patients (9.8%) received consolidation therapy after the frontline ASCT: 3/5 patients received lenalidomide, dexamethasone (Rd) and 2/5 patients received VTD. Nine patients (17.7%) received maintenance therapy following the frontline ASCT: 5/9 patients received lenalidomide, 2/9 patients thalidomide, 1/9 patient interferon and 1/9 patient ixazomib. In total, 7 patients (13.7%) were previously exposed to lenalidomide during induction, consolidation or maintenance of first line of therapy, but none of them were refractory to lenalidomide. The median interval between the start of the first and second line of treatment was 40.2 months (IQR 30.9–53.4), while the median interval between the first and ASCT was 40.4 months (IQR 31.7–55.1). The median interval between the start of the second line treatment and the transplant was 5.9 months (IQR 4.8–8.1). Regarding the number of cycles of KRd received in induction, 24 patients received 3 or 4 cycles (50.0%), 16 patients five or six cycles (33.3%) and 8 patients seven to twelve cycles (16.7%), data was missing for 3 patients. Carfilzomib administration schedules were biweekly in 24 patients (68.6%) and weekly in 11 patients (31.4%), data was missing in 16 patients. Regarding conditioning for the second line ASCT, the majority underwent melphalan conditioning alone (*n* = 46; 90.2%), melphalan and bortezomib (*n* = 2; 3.9%), melphalan and bendamustine (*n* = 1; 2.0%), melphalan and busulfan (*n* = 1; 2.0%) and cyclophosphamide (*n* = 1; 2.0%). Following the ASCT, 9 patients received consolidation chemotherapy: 7/9 patients received KRd and 2/9 ixazomib, lenalidomide, and dexamethasone. Twenty-three patients received maintenance chemotherapy: 18/23 lenalidomide, 2/23 pomalidomide, 1/23 bortezomib, 1/23 ixazomib and 1/23 thalidomide. Six patients received an allogeneic stem cell transplant (alloSCT) following the second line ASCT as consolidation.

**Table 1:** Population description.

	Total (*n* = 51)
Age (years) – median (IQR)	61 (58–66)
≥ 60 years – *n*(%)	35 (68.6)
≥ 65 years – *n*(%)	19 (37.3)
Sex (male) – *n*(%)	35 (68.6)
Monoclonal component – *n*(%)	
IgG	29 (56.9)
IgA	12 (23.5)
Free light chain only	10 (19.6)
ISS disease stage – *n*(%)	
I	18 (42)
II	11 (26)
III	14 (32)
Missing	8
Cytogenetic profile risk – *n*(%)	
Standard risk	27 (71)
High risk	11 (29)
del(17p)	6 (16)
t(4;14)	5 (13)
Missing	13
Patients exposed to lenalidomide in 1st line of therapy – *n*(%)	7 (13.7)
Time between start of 1st and 2nd line of therapy (month) – median (IQR)	40.2 (30.9–53.4)
Time between 1st and 2nd ASCT (month) – median (IQR)	40.4 (31.7–55.1)
Time between start of 2nd line of therapy and 2nd ASCT (months) – median (IQR)	5.9 (4.8–8.1)
Number of cycles of KRd in induction – n (%)	
3 or 4	24 (50.0)
5 or 6	16 (33.3)
7 to 12	8 (16.7)
Missing	3

### Overall response rate (ORR), progression-free survival (PFS) and overall survival (OS)

**Fig. 1:**
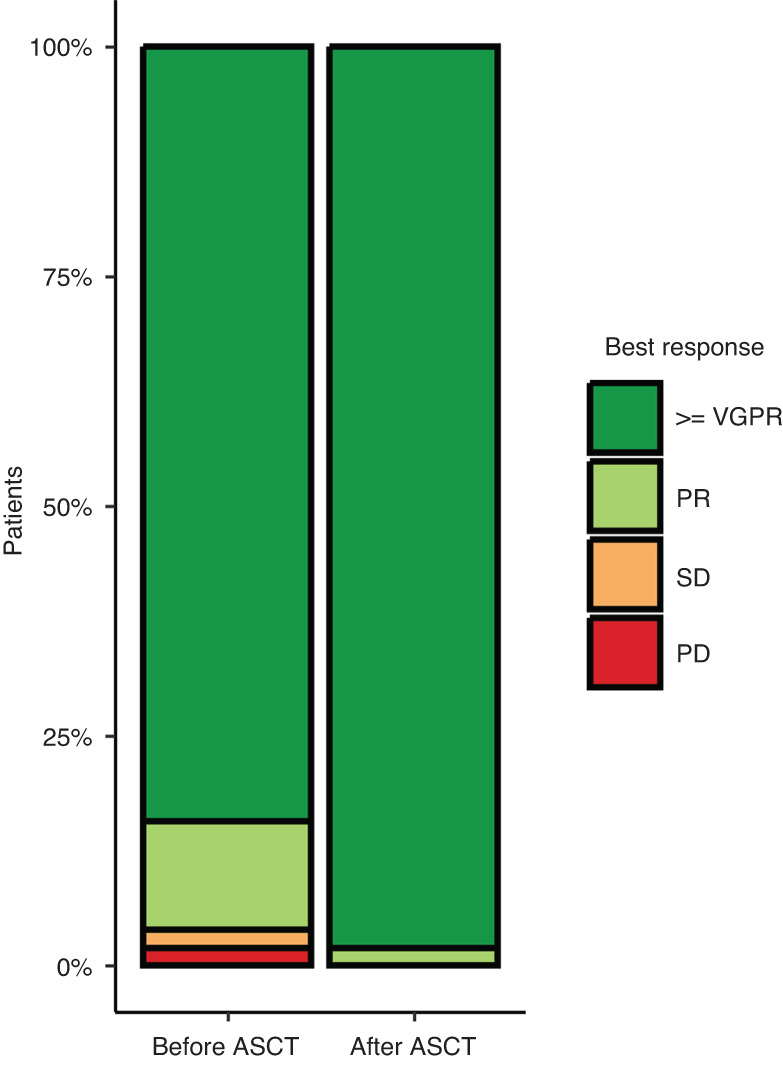
Overall response rates. Best observed responses, before and after the second ASCT.

**Fig. 2:**
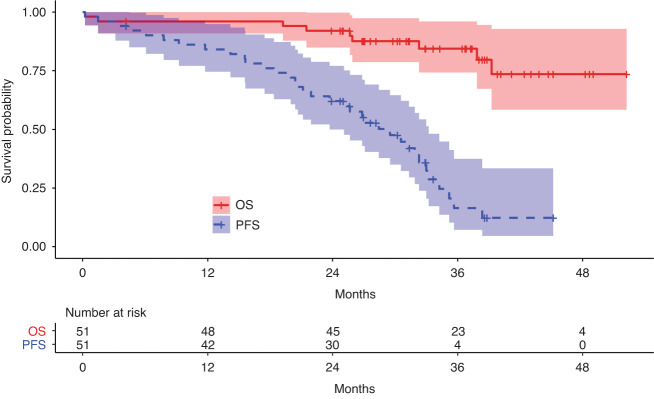
Survival assessment. Progression-free survival (PFS) in blue and overall survival (OS) in red. The shaded areas represent the 95% confidence interval.

The best observed treatment responses, before and after ASCT, are shown in Fig. 1. ORR was 96% before ASCT and 100% after ASCT, with 43 patients (84.3%) achieving very good partial response (VGPR) or better before ASCT and 50 patients (98%) after ASCT. The median follow-up was 36.7 months (range 0.2 to 45.2 months). The median PFS was 29.5 months (IQR 18.4–34.2) and the median OS was not reached (Fig. 2). The OS probability rate was 96.1% (90.1–100) at 12 months, 92.1% (84.9–99.8) at 24 months and 84.5% (74.3–96.0) at 36 months. During the follow-up period, 26 patients relapsed and nine deaths were reported. Of these, two deaths were of infectious origin immediately after second line ASCT, two were due to late infections at 21.5 and 41.7 months after ASCT, one died due to progression, one died of an "other cause” and three deaths occurred due to infection following a subsequent alloSCT.

### Factors influencing PFS and OS

**Fig. 3:**
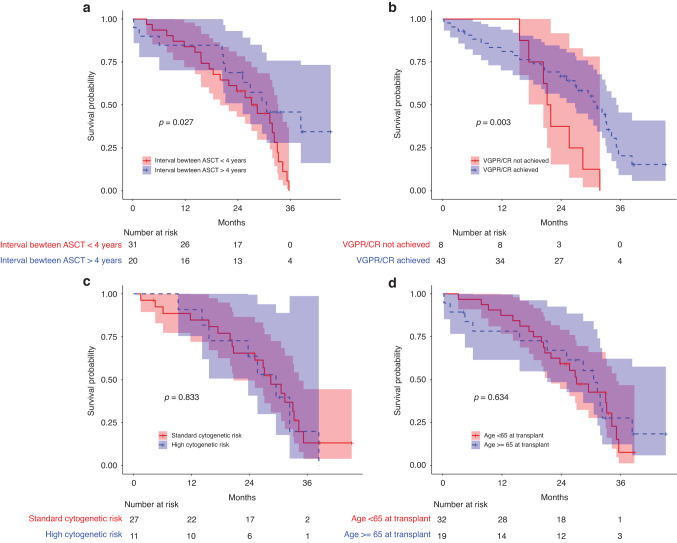
PFS per subgroups. PFS - subgroup analyses according to time between transplants (**a**), achievement of VGPR or CR before the 2. ASCT (**b**), cytogenetic risk (**c**) and age at transplant (**d**). Shaded areas represent the 95% confidence interval and the *p*-values correspond to the results of the mdir log-rank test.

Univariate analyses of co-factors influencing PFS is summarized in Table 2. A significant association was observed between the time interval (in months) between transplants and PFS in our study (parameter estimate = −0.02521, *p* = 0.0416). Stratifying the data, we identified a favorable outcome for patients with up to 4 years between transplants (*p* = 0.027, Fig. 3a), where the median PFS was 30.6 months compared to 28.4 months. Furthermore, achieving a VGPR or better prior to ASCT was also found to be significantly associated with improved PFS (*p* = 0.003, Fig. 3b), with a median PFS of 31.3 months compared to 21.5 months. However, no statistical association was found between PFS distributions and cytogenetic risk profile (Fig. 3c), age > 65 years (Fig. 3d), ISS score, depth of response to the first ASCT, exposure to lenalidomide at first line of treatment, number of KRd cycles received in induction, administration schedule, consolidation therapy, maintenance therapy or alloSCT following ASCT. Concerning OS, no significantly relevant co-variables were found in univariate analysis, including an interval between frontline and relapse ASCT of more than 4 years, the achievement of a VGPR or better before the 2nd ASCT or a cytogenetic high-risk profile.

**Table 2:** Univariate analyses of co-factors influencing PFS.

	Median PFS (95% IC)	*p*-value
Age		0.63
< 65	27.1 months (21.9–35.2)	
> 65	30.6 months (21.2–NA)	
Score ISS III		0.95
I/II	31.9 months (23.7–38.4)	
III	27.1 months (21.2–NA)	
High-Risk cytogenetic profile		0.83
No	28.4 months (20.7–34.2)	
Yes	29.5 months (23.7–NA)	
VGPR / CR achieved after 1st ASCT		0.12
No	34.2 months (33.0–NA)	
Yes	27.1 months (21.9–32.3)	
Previous exposure to lenalidomide		0.55
No	28.4 months (23.7–33.0)	
Yes	35.7 months (20.4–NA)	
Number of KRd cycles administered		0.83
3–4	29.5 months (21.9–NA)	
5–6	30.6 months (25.1–NA)	
7–12	29.0 months (21.2–NA)	
Carfilzomib administration scheme		0.83
Bi-weekly	26.0 months (20.0–NA)	
Weekly	30.6 months (20.4–NA)	
**VGPR / CR achieved before 2nd** **ASCT**		**0.003**
**No**	**21.5 months (20.40**–**NA)**	
**Yes**	**31.3 months (26.8–35.7)**	
**Time** > **4 years between transplants**		**0.027**
**<** **4 years**	**28.4 months (20.0–33.2)**	
**>** **4 years**	**30.6 months (25.10**–**NA)**	
Allogenic transplant before relapse		0.36
No	29.5 months (25.1–34.2)	
Yes	24.7 months (9.2–NA)	
Consolidation therapy given		0.45
No	27.1 months (21.9–33.0)	
Yes	35.2 months (21.2–NA)	
Maintenance therapy given		0.13
No	25.7 months (18.4–38.4)	
Yes	30.6 months (27.1–NA)	

Significant differences are placed in bold.

## Discussion

This retrospective, multicenter, international study investigated characteristics and outcome of 51 MM patients treated with KRd followed by a second ASCT in their first relapse. The median PFS in this population was 29.5 months and the median OS was not reached. We report a treatment-related mortality (TRM) of 4%, with two patients dying within days of the procedure as a result of infectious complications. This TRM rate is higher than reported in frontline ASCT. However, drawing firm conclusions is difficult given the limited number of patients, as well as the multicentric and international nature of this study. In univariate analyses, we found that achievement of a VGPR or better before the second ASCT and/or more than four years between frontline and relapse ASCT was associated with a longer median PFS. No statistically significant factor was found for OS. This study confirms the value of this treatment sequence in MM patients at first relapse.

There are currently three second-line treatment options recommended by the EHA/ESMO and IMWG for patients with lenalidomide-sensitive disease: daratumumab, lenalidomide, dexamethasone (DRd), carfilzomib, lenalidomide, dexamethasone (KRd) or ixazomib, lenalidomide, dexamethasone (IRd). It is notable that in the ASPIRE trial comparing KRd to Rd, the median PFS was 26.3 months in patients treated with KRd who had received 1 to 3 lines of treatment and 29.6 months at first relapse [8]. A ‘real world’, retrospective study by the Rete Ematologica Pugliese (REP) of 130 patients treated with KRd without ASCT for first relapse of MM found a median PFS of 24 months and a median OS of 33 months [17]. Another retrospective real-world study reporting the outcomes of 44 patients treated with KRd + ASCT in Heidelberg, Germany, who received 1 to 3 lines of treatment including a first ASCT, showed a PFS of 23 months after the second ASCT. They identified response status at the time of transplantation and maintenance therapy as having a prognostic impact on progression-free survival (PFS) [18]. In the TOURMALINE-MM1 trial, comparing IRd to Rd in patients who had received one to three prior lines of treatment, the median PFS was 20.6 months [19]. Finally, in the POLLUX trial comparing DRd to Rd, the median PFS was 44.5 months with DRd in patients who had received one to three prior lines of treatment and 53.3 months for patients with only one prior line of treatment [20].

As opposed to the ASPIRE and POLLUX studies, our starting point is the day of ASCT and not the day of treatment initiation. Another point is that only 47% of patients in our cohort received a maintenance therapy, while all patients had a continuous treatment in ASPIRE and POLLUX studies. Altogether, DRd remains the preferred treatment option in patients with lenalidomide naïve first relapse [21]. A randomized controlled phase III trial by the German Multiple Myeloma Group (GMMG) comparing Rd + ASCT to continuous Rd in first to third relapse did not show a PFS (21 vs. 19 months) or OS (not reached vs. 63 months) benefit, but almost 30% of patients did not receive the allocated transplant due to side effects or disease progression [22]. A prospective, randomized trial would therefore be needed to assess the efficacy of intensified first relapse MM therapy in combination with state-of-the-art triplet induction regimens, with or without maintenance therapies.

We chose the day of ASCT as the starting point for this study because, using the EBMT database, we identify patients who actually received an ASCT but cannot identify patients who were scheduled to receive one but who did not proceed to ASCT for any reason (i.e., infections or relapse). Starting our analyses on the first day of KRd would introduce a major bias. Similarly, exact melphalan doses prescribed, specific toxicities of KRd and occurrence of secondary myeloid neoplasms were not prospectively reported.

In our analysis, the best outcomes were seen in patients who achieved a VGPR or better before ASCT, independently of the number of cycles of KRd in induction, and for patients with a long interval between the two ASCTs. As seen previously, the various international recommendations suggest an interval of 18 months to three years between the two ASCTs, but our study found a statistically significant benefit beyond four years. Interestingly, there was no difference in OS or PFS between patients with standard and high-risk cytogenetic profiles in our cohort. This supports the idea that treatment intensification helps to improve the prognosis of patients with high-risk cytogenetic MM. However, this is a subgroup analysis with small sample sizes. In addition, we could not apply a cut-off for positivity for the detection of del(17p), the results of which were center-dependent.

In the study, consolidation and maintenance treatment after the second ASCT did not affect OS or PFS. However, we do not have information on the exact duration of these treatments or the discontinuation rate so any interpretation can only be provisional. In addition, a small subset of patients was exposed to lenalidomide in the first-line regimen and did not have a statistically different OS or PFS distribution. Finally, it is interesting to note that six patients received an alloSCT following their ASCT. In the univariate analysis, there was no significant benefit on PFS or OS and two patients died within 100 days of transplant but our sample size is too small to draw any conclusion.

According to the IMWG criteria, a bone marrow sample with a plasma cell count < 10% is required to confirm complete response [23]. As this study was retrospective, this information was missing in most cases; we therefore chose to combine VGPR or better responses.

Despite inherent limitations, our study identifies some variables that can aid in the selection of patients who are most likely to benefit from a second ASCT. This is clinically relevant information due to the lack of randomized clinical trials assessing this approach. While a majority of MM patients do not undergo a second ASCT, it still remains an interesting option in the early stages of the disease, and a subgroup of patients may derive significant benefits from this procedure. There is a need for well-defined criteria to identify these patients given the increasing number of treatment options at relapse and the advent of new-generation therapies such as CAR-T cells and bispecific antibodies. Finally, in countries with limited access to new generation therapies a second ASCT represents an efficient treatment option. In conclusion, KRd followed by an ASCT is an effective treatment option for transplant-eligible patients at first relapse. It should be particularly considered in patients with more than four years between frontline and relapse ASCT and/or who achieved at least a VGPR before the second ASCT.

## Data Availability

The data that support the findings of this study are available on request from the corresponding author. The data are not publicly available due to privacy or ethical restrictions.
